# Experimental and numerical study on the model of hybrid fiber phase change concrete frozen shaft wall

**DOI:** 10.1371/journal.pone.0306984

**Published:** 2024-08-08

**Authors:** Dongwei Li, Zhiwen Jia, Zecheng Wang, Kaixi Xue, Zhenhua Wang, Fang Fang

**Affiliations:** 1 College of Civil Engineering and Architecture, Dalian University, Dalian, China; 2 School of Civil and Architectural Engineering, East China University of Technology, Nanchang, China; Jamia Millia Islamia, INDIA

## Abstract

In this study, phase change materials (PCMs) were innovatively incorporated into hybrid fiber concrete. The properties of PCMs, which absorb and release heat during phase transitions, enable the concrete to actively respond to complex and varying temperature environments. This integration reduces the internal temperature differentials within the concrete, thereby preventing temperature-induced cracks in deep wellbore structures. Through the temperature control model test of the frozen shaft wall, it can be seen that the hybrid fiber phase change concrete (HFPCC) significantly reduces the internal temperature difference, and the maximum temperature difference along the radial direction is 35.84% lower than that of benchmark concrete (BC). The numerical simulation results indicate that a moderate phase transition temperature should be selected in engineering. The phase change temperature should not be close to the ambient temperature and peak temperature. The peak temperature can be reduced by 9.32% and the maximum radial temperature difference can be reduced by 30.89% by selecting an appropriate phase change temperature. The peak temperature and radial maximum temperature difference are both proportional to the latent heat of phase change. The temperature control performance of phase change concrete can be further improved by increasing the latent heat of phase change materials.

## 1. Introduction

In order to resist the strong ground pressure and water pressure, the early strength and high strength mass concrete shaft lining structure is usually adopted in the design of frozen shaft lining structure in deep soil layer. The thickness of inner and outer shaft lining is large [1, 2]. Upon pouring the concrete for the shaft wall, the initial reaction is vigorous, leading to rapid temperature fluctuations and increased hydration temperature. The low thermal conductivity of the shaft wall, compounded by external environmental factors, results in significant temperature differentials between the interior and exterior of the structure, thereby precipitating the formation of temperature-induced cracks and posing numerous risks to the safe construction and utilization of the shaft wall [3, 4]. At present, the research on the mechanism of wellbore temperature cracks mainly focuses on temperature field and temperature stress. Zhang et al. [5], Yu et al. [6], and Li et al. [7] have established models of the early temperature field and stress field in frozen wellbore walls based on the theory of thermoelasticity. They combined on-site measured data to analyze the evolution laws of the temperature field and stress field, and found that significant temperature stress forms in the early stage of frozen wellbore walls, posing a risk of fracture.

The damage of mass concrete structure is closely related to the hydration heat of concrete and the complex external environment. After long-term construction and research accumulation, a variety of measures to prevent concrete temperature cracks have been produced. Traditional measures mainly include raw material selection and optimization of mix ratio [8–10], raw material precooling [11, 12], improvement of construction technology [13] and maintenance temperature control [14]. These measures can reduce the hydration temperature rise of mass concrete to a certain extent and avoid the occurrence of temperature cracks, but the preparation work is cumbersome, the construction process is complex and the cost is high, which is not suitable for freezing shaft lining concrete. In addition, scholars have added concrete admixtures to control the crack problem of concrete structures. The expansion agent can undergo a hydration reaction, crystallize at the crack, and produce a self-healing effect [15, 16]; the superplasticizer can reduce the amount of water in concrete, thereby reducing the heat of hydration reaction and the thermal stress of concrete [17, 18]; the temperature rise inhibitor is adsorbed on the surface of C-S-H gel, which inhibits the further growth of the gel and reduces the hydration temperature rise [19–21]. Adding admixtures has a certain stress control effect, but the control effect is not good for special environments, especially in the case of high adiabatic temperature rise.

Phase change concrete is a new type of functional building material with a high latent heat of phase change [22]. Researchers utilize the properties of phase change concrete to absorb (release) a significant amount of heat during the phase change process, proactively addressing complex temperature environments and their fluctuations. This helps reduce the internal temperature difference of concrete, thereby preventing the occurrence of temperature-induced cracks in mass concrete structures under complex conditions [23–25]. Kim et al. [26] selected barium-based PCM as a latent heat adhesive. Test results demonstrate that barium-based PCM effectively regulates the hydration heat in mass concrete, maintaining the maximum temperature of the structural core at approximately 58°C. Shi and Hou [27] directly added paraffin emulsion into high-volume concrete, resulting in a significant decrease in concrete temperature rise. Additionally, when environmental temperatures fluctuated, the concrete temperature exhibited more gentle variations. The incorporation of phase change materials can enhance the heat storage and temperature regulation capabilities of concrete. Nevertheless, the limitations of the preparation process result in low strength of phase change materials or phase change aggregates, thereby exerting a significant adverse effect on the mechanical properties of concrete [28–30]. The direct mixing of high-strength materials, fiber materials, etc. with cement-based materials can significantly improve their mechanical properties. Xue et al. [31] found that the addition of 10% steel slag fine aggregate (SSA) in concrete specimens resulted in the lowest porosity and an ideal pore structure, with a 5.60% increase in compressive strength. Gürbüz and Erdem [32] studied the addition of hybrid fibers (0.5% steel fibers and 1.5% polyethylene fibers) in phase change concrete. The hybrid fibers restricted the surrounding matrix material at a microscopic scale, resulting in a triaxial compression state. Due to this mechanism, the compressive strength loss in phase change concrete was significantly compensated, and the flexural strength increased.

Although there has been some progress in the research of phase change materials in the field of thermal control for large volume concrete, there is little research on phase change concrete for freezing well walls, and its application effectiveness and feasibility have not been fully determined. In this study, hybrid fiber phase change concrete (HFPCC) is prepared to simplify the heat transfer process of frozen shaft wall, and the temperature control model test of frozen shaft wall is carried out and the numerical simulation analysis is carried out. The research unveils the temporal and spatial temperature field variations in the shaft wall, and investigates the impact of integrating phase change materials into the frozen shaft wall structure for temperature control. Furthermore, it delves into the influence of material parameters on the temperature control efficacy of phase change concrete, offering insights to enhance the utilization of these materials.

## 2. Model test

This experiment studies the temperature field of benchmark concrete (BC) and phase change fiber reinforced concrete well walls, reveals the development law of well wall temperature over time, explores the temperature control effect of adding phase change materials to concrete structures, and conducts feasibility analysis.

### 2.1 Materials

An ordinary Portland cement with a grade of 42.5R was used to prepare concrete in this study. Fly ash is a gray powder with a density of 2.55. Silica powder is grayish white powder with a specific surface area of 21m^2^/g. The river sand was utilized for the fine aggregates, its fineness modulus is 2.48. The continuously graded natural crushed stone was adopted for the coarse aggregates with a particle size range of 5mm~20mm. The basic properties of expanded perlite, as measured in the test, are shown in Table 1. The phase change materials used include 48# fully refined paraffin wax and 5# liquid phase change wax. The properties of these materials are detailed in Table 2. Furthermore, the ordinary tap water and high-efficiency water-reducing agent were also adopted in this study.

**Table 1:** Basic properties of expanded perlite.

Grain size (mm)	Thermal conductivity (W/(m·k))	Bulk density (kg/m^3^)	Apparent density (kg/m^3^)	Compressive strength of concrete cylinder (MPa)	1h water absorption (%)
1.18–4.75	0.025~0.049	160.6	408.3	0.418	74.3%

**Table 2:** Properties of paraffin.

Phase change material	*T*_0_ (°C)	*T*_*e*_ (°C)	*T*_*p*_ (°C)	ΔH (J/g)
5#	1.56	12.17	5.97	200.84
48#	21.70	36.11	27.91	30.57
	41.59	56.18	50.50	136.78
Binary phase change paraffin	0.21	5.25	2.58	66.52
	34.17	49.92	44.52	102.63

Note: *T*_0_ is the initial melting temperature, *T*_*e*_ is the complete melting temperature, *T*_*p*_ is the Peak temperature of phase transition, and ΔH is the latent heat of phase change.

### 2.2 Preparation of phase change aggregate

**Fig 1:**
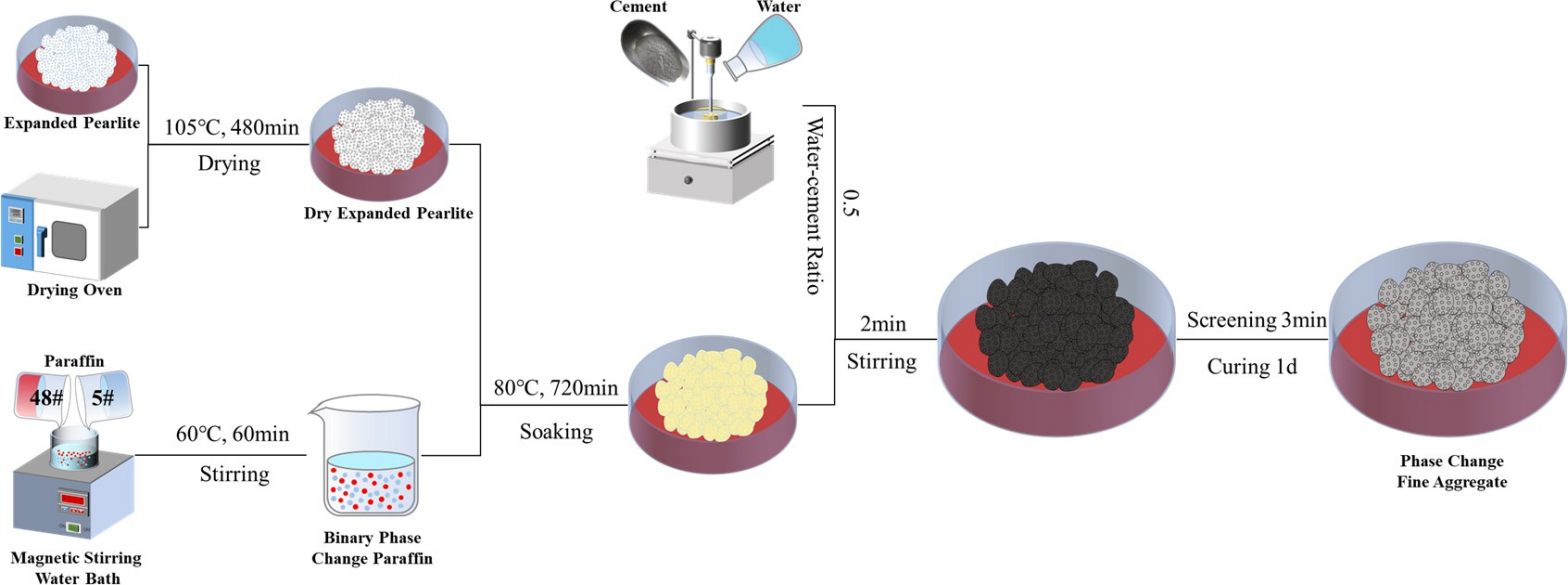
Preparation flow chart of phase change fine aggregate.

The binary melting phase change paraffin wax was prepared using the melt blending method, as shown in Fig 1. A certain amount of melted 48# paraffin wax and 5# paraffin wax (mass ratio 7:3) were weighed in the same beaker and placed in a constant temperature water bath (60°C) for 60 minutes and stirred to ensure that the two kinds of paraffin wax were fully mixed. Expanded perlite was chosen as the carrier material, demonstrating an adsorption rate of 108.58%. Subsequently, the expanded perlite, which had adsorbed a phase change material, was enclosed within cement paste. Notably, the density of the phase change perlite increased from 0.850 g/cm^3^ to 1.036 g/cm^3^ following encapsulation.

### 2.3 Preparation of phase change aggregate

The BC mix ratio is marked as C50, and the mix ratio designs are carried out in accordance with the China JDJ 55–2011 specification. The water-cement ratio is 0.35, the sand ratio is 36%, and the dosage of water reducing agent is 1% (based on cementitious materials). The HFPCC uses 50% of the phase change aggregate to replace the fine aggregate in the concrete, the steel fiber content is 0.903%, and the carbon fiber content is 0.293%. The concrete mix is shown in Table 3.

**Table 3:** Mix proportions of concrete unit: kg/m^3^.

Specimen	Cement	Flyash	Silica fume	Water reducing agent	Aggregate	Sand	Water	Phase change aggregate	Steel fiber	Carbon fiber
BC	385	110	55	5.5	1089.3	612.7	192.5	0	0	0
HFPCC	385	110	55	5.5	1089.3	306.4	192.5	132.9	70.4	5.2

### 2.4 Test scheme

Phase change concrete has yet to be utilized in frozen wellbore structures. The prototype testing requires a substantial amount of testing materials and a series of engineering equipment to gather experimental data. As a result, scaled-down model tests are conducted in experimental research.

#### 2.4.1 Model assumptions

In this experiment, the scale test of frozen shaft lining is designed, and the following assumptions are made:

1. It is considered that the heat transfer inside the concrete is only transmitted along the radial direction, and the vertical heat transfer is not considered.
2. The temperature field of freezing shaft wall is uniform along the circumferential direction.

**Fig 2:**
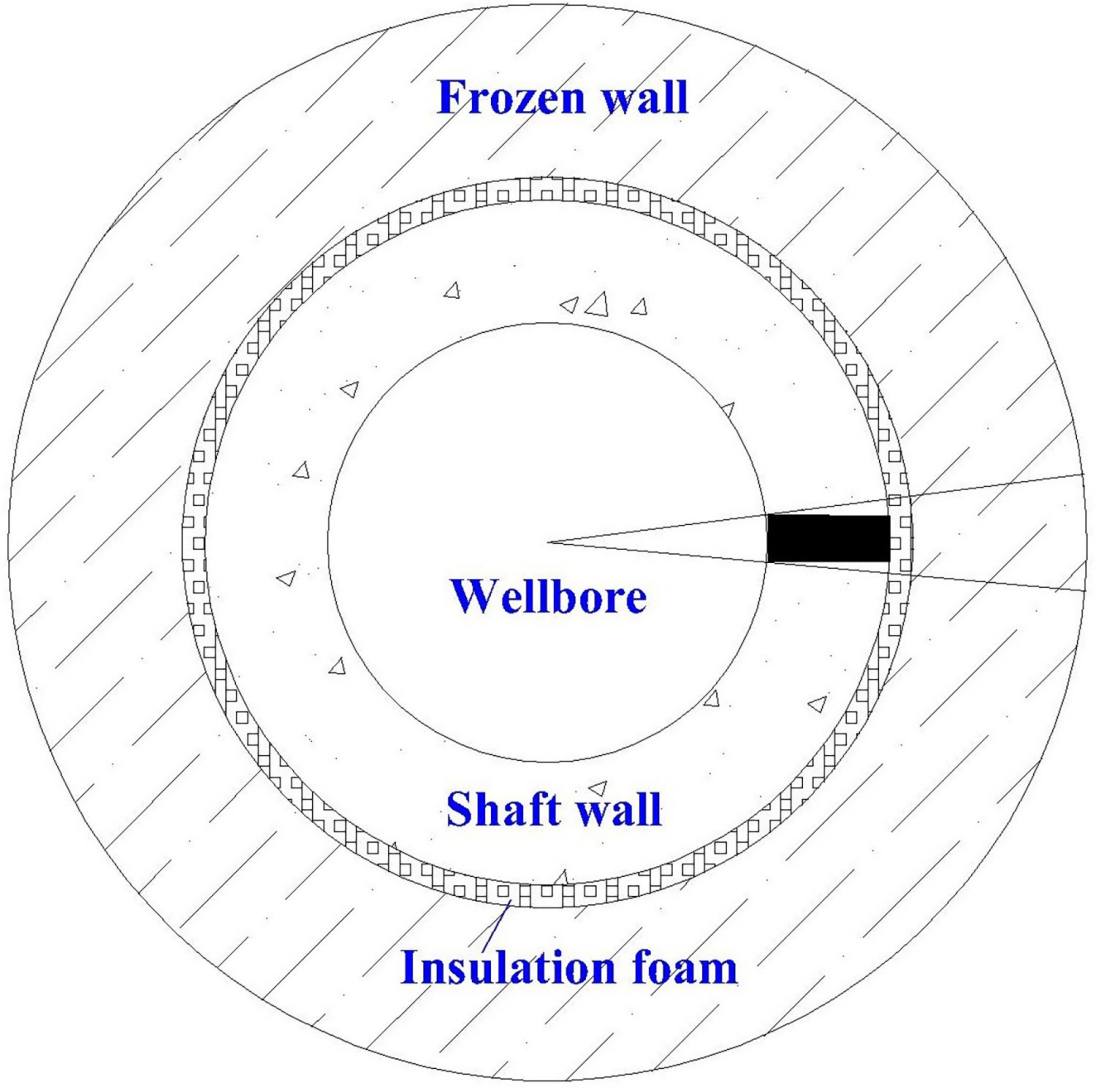
Schematic diagram of the well wall.

Through the above assumptions, the wellbore temperature field model can be selected at any angle, as shown in Fig 2. The study found that the wellbore model can be simplified to a rectangle (Fig 2 Shadow) [33]. The test model needs to meet the following boundary conditions:

1. Due to the assumption of radial heat transfer, the vertical surfaces are thermally insulated.
2. Due to the assumption of a uniform temperature field along the circumferential direction within the wellbore and the absence of heat transfer in the circumferential direction, insulation is applied to both sides.
3. Modeling interior wall interactions with air interface.

#### 2.4.2 Design of temperature control model box

**Fig 3:**
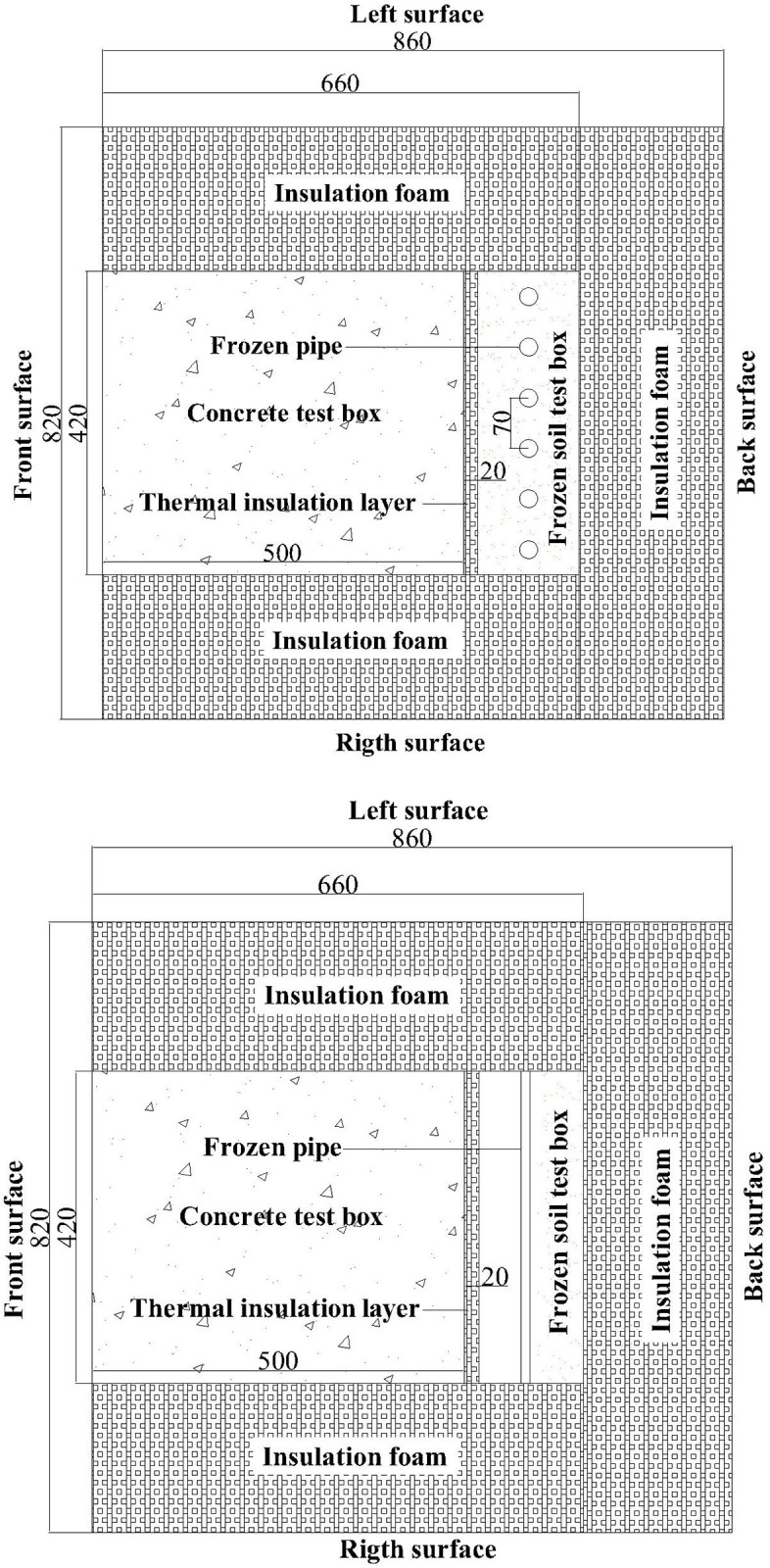
Schematic diagram of concrete model test chamber.

The temperature control model box is composed of frozen soil test box, the insulation layer and concrete test box, as shown in Fig 3. The total size of the model box is 860mm×820mm×820mm (length×width×height), in which the size of the frozen soil test box is 140mm×420mm×420mm (length×width×height), the insulation layer is 20mm foam board, the size of the concrete test box is 500mm×450mm×450mm (length×width×height, corresponding to the radial thickness, arc length and height of the shaft wall), and the box is surrounded by 200mm thick insulation foam for adiabatic treatment.

**Fig 4:**
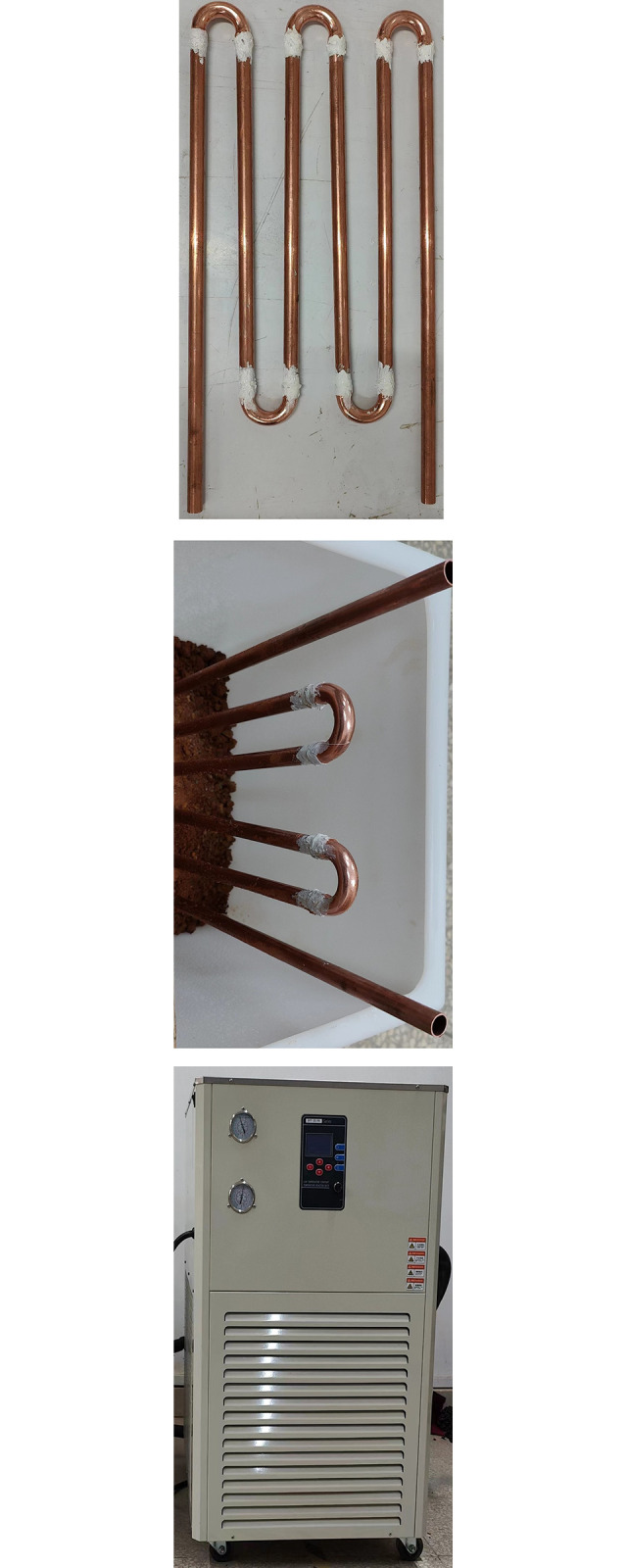
Freeze the test chamber system.

The frozen soil test box is equipped with red clay with a water content of 20% and a dry density of 1.69 kg/m^3^. Fig 4 shows the arrangement of freezing pipes, the φ12.5×0.8 copper pipe is used as the freezing pipe, and the interval between the freezing tubes is 70 mm.

The thermal insulation layer consists of a 20 mm foam board that is effectively integrated with both the frozen wall and the shaft wall.

**Fig 5:**
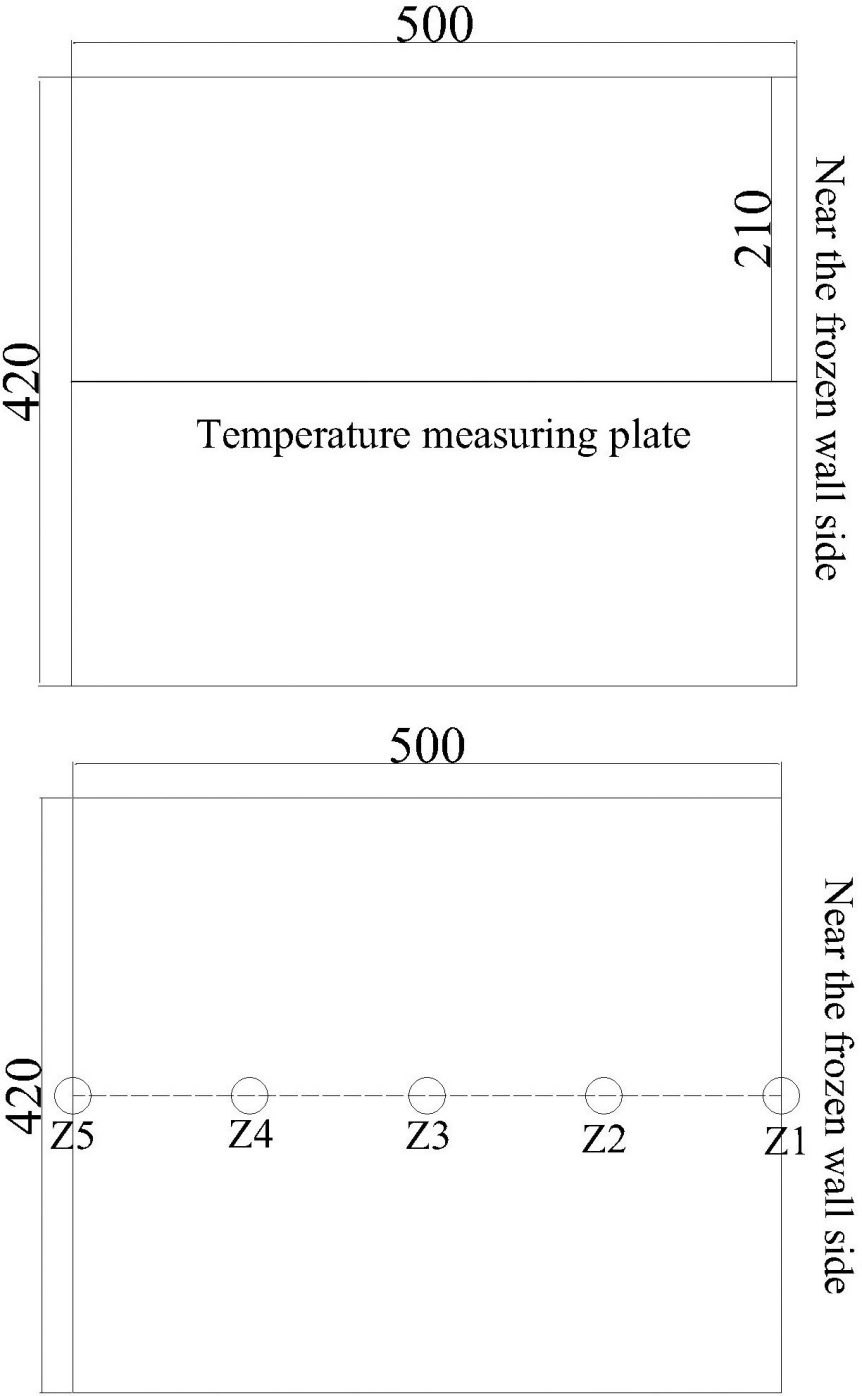
Schematic diagram of the temperature sensor arrangement.

The concrete test box is equipped with different proportions of concrete. The temperature acquisition adopts SH-16 X multi-channel temperature recorder. The sensor adopts K-type thermocouple, and the measurement accuracy is ± 0.1. According to the temperature measurement position, the temperature sensor is fixed on the temperature measurement plate, and then the temperature measurement plate is placed in the concrete test box. The sensor layout diagram is shown in Fig 5.

**Fig 6:**
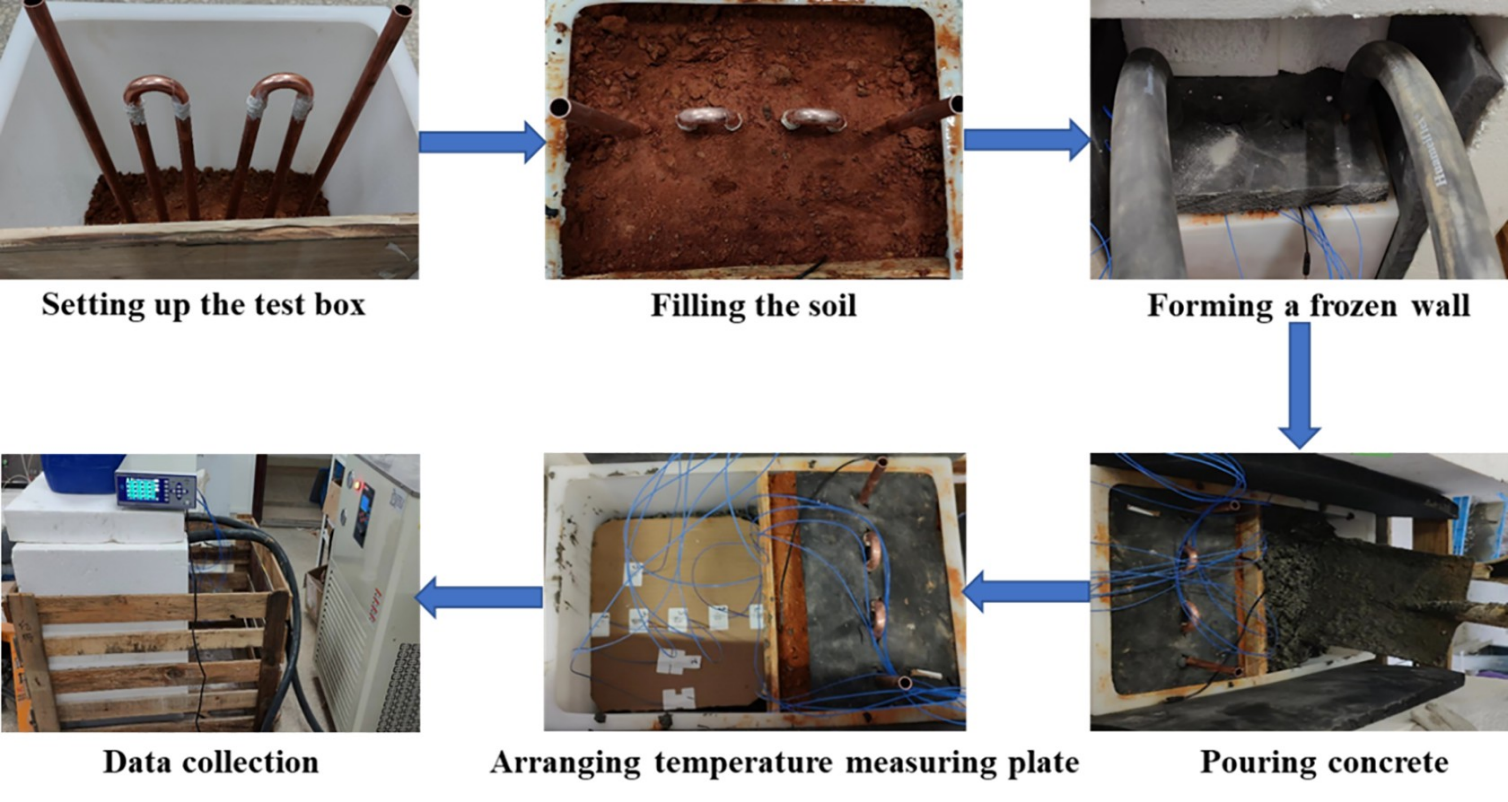
The process of temperature control model test.

The test process is shown in Fig 6. The specific steps are as follows:

1. Setting up the test box: Construct the test box according to the model design scheme, including frozen soil test box, concrete test box, thermal insulation layer, insulation foam.
2. Forming a frozen wall: Install the freezing pipes, fill with soil, and compact it; connect the cold bath circulation system, start the refrigeration system, and monitor the temperature of the frozen wall in real time, reducing it to -2°C.
3. Pouring concrete: Mix the concrete mixture according to the mix ratio and divide the wellbore into two layers from the height. The experimental process is as follows: pour the first layer of concrete—vibrate and level—arrange the temperature measurement board—pour the second layer of concrete—vibrate and level. After the concrete is completed, cover it with an insulation layer.
4. Data Collection: Connect the temperature data logger with a data collection frequency of 30 seconds per reading.

### 2.5 Experimental results analysis

#### 2.5.1 The variation of temperature with time

**Fig 7:**
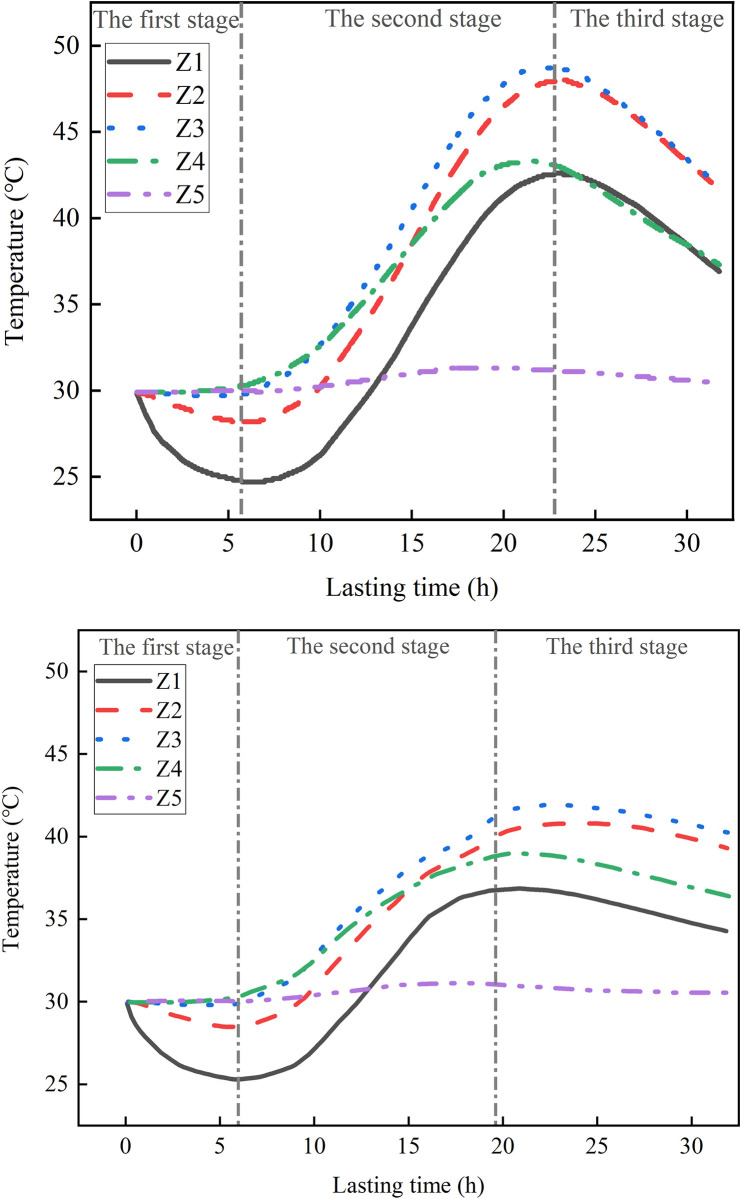
Temperature rise curve.

**Fig 8:**
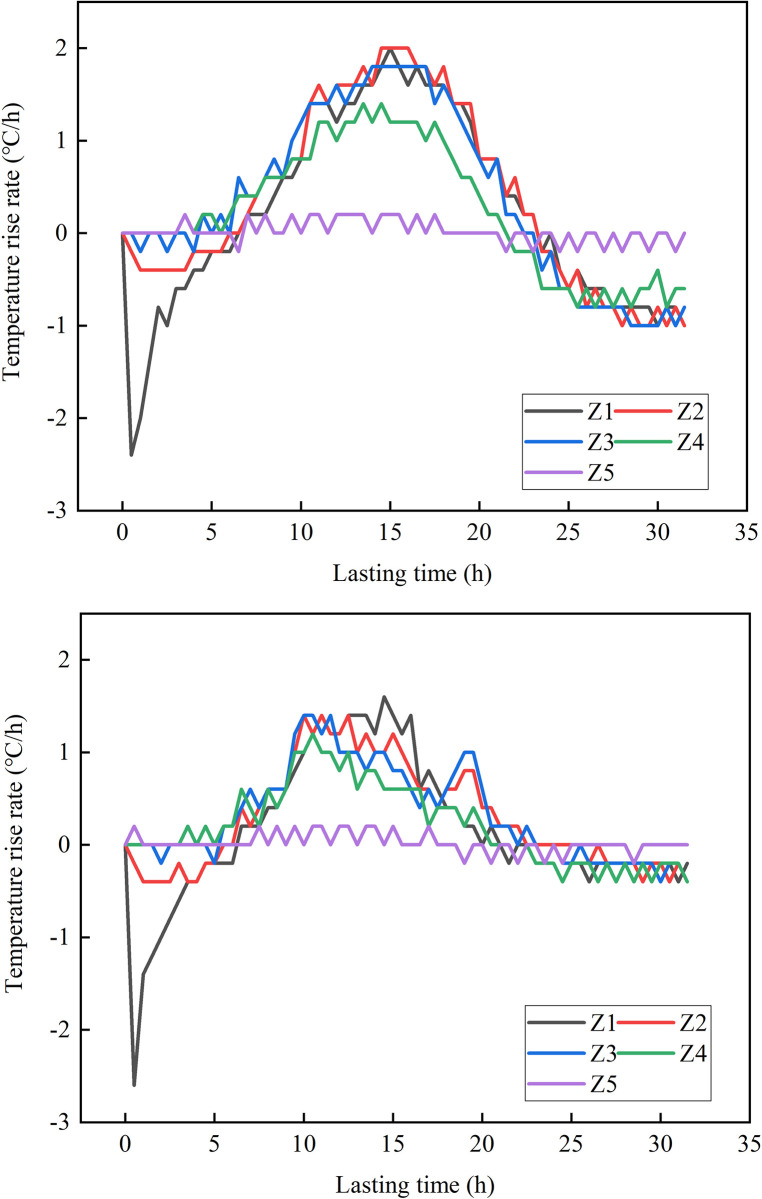
Temperature change rate curve.

Fig 7 shows the change rule of temperature of measuring points Z1~Z5 with time, and Fig 8 shows the change rule of temperature change rate of measuring points Z1~Z5 with time. The measured data are presented in S1 Table. The temperature of each measuring point can be divided into three stages after concrete pouring. Taking the BC Z1 as an example, the first stage: in the period of 0~5.71h, the temperature shows a downward trend with the increase of time, and the change rate is negative at this time. The temperature of the measuring point near the side of the shaft wall decreases more obviously. At this stage, the heat release of concrete belongs to the induction period, and the temperature of the shaft wall is affected by the frozen wall. The second stage: within 5.71~22.78h, the hydration reaction is severe, the temperature rises rapidly, the temperature rise rate is large, the temperature rise is fast, and the temperature reaches the peak value at this stage; The third stage: after 22.78h, the temperature decreases rapidly, and the temperature rise rate gradually decreases and remains constant at-1.0°C/h.

In the first stage, the temperature rise curve and temperature rise rate curve of the two kinds of concrete are consistent. In the second stage, the curve shows significant differences. The peak temperatures for Z1 to Z5 in the BC were 42.6°C, 48.0°C, 48.7°C, 43.4°C, and 31.3°C, respectively. In comparison, the peak temperature of the HFPCC was consistently lower than that of the standard concrete, with reductions of 5.8°C, 7.3°C, 6.8°C, 4.5°C, and 0.2°C for Z1 to Z5, respectively. Prior to reaching the maximum rate of temperature increase (10~12h) in the HFPCC, the temperature rise rate curves of both materials exhibit a fundamental similarity. Subsequently, the temperature rise rate of the HFPCC gradually declines, whereas the temperature rise rate of the BC continues to ascend, peaking between 13 and 15 hours. This clearly demonstrates that when phase change materials are added to concrete, they have a significant impact in lowering its temperature and delaying the rate at which the temperature rises. During the third phase, the temperature of the BC decreases rapidly, and the cooling rate is high. On the other hand, the temperature of the HFPCC drops gradually, and the cooling rate decreases gradually until it reaches a constant value of -0.4°C/h. This clearly demonstrates that the incorporation of phase change materials effectively mitigates the rate at which concrete cools down.

#### 2.5.2 Temperature distribution rule

**Fig 9:**
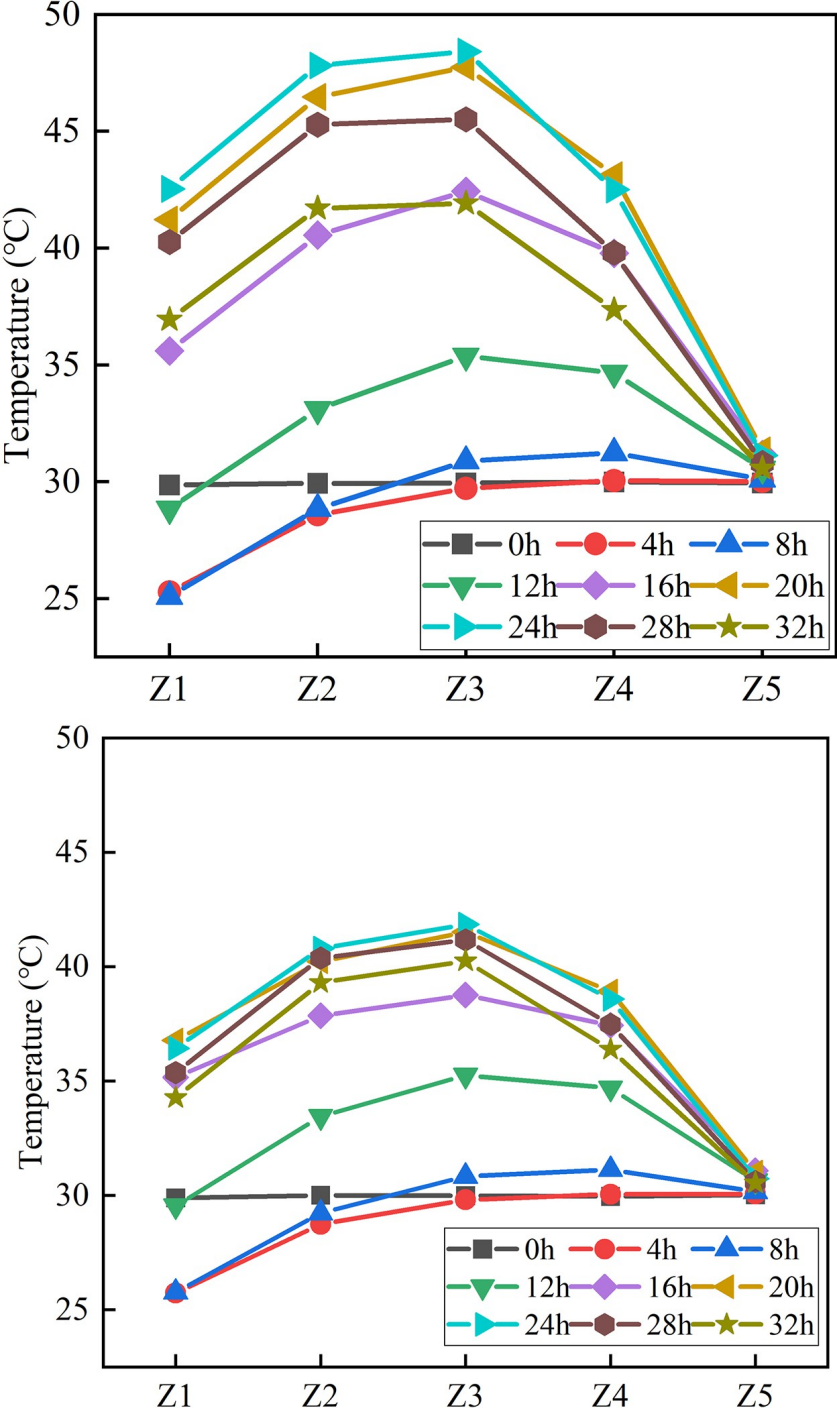
Temperature distribution law of Z1~Z5 measurement point direction.

Fig 9 shows the temperature distribution of concrete Z1~Z5 at different time. In the wellbore model, the temperature varies in a parabolic manner along the radial direction. The central temperature is elevated, while the temperature on both sides is comparatively lower. The radial temperature change of HFPCC is slower than that of BC. The BC exhibits varying radial temperature differences at different time intervals, with values of 0.1°C, 4.8°C, 6.1°C, 6.5°C, 11.3°C, 16.4°C, 17.3°C, 14.7°C, and 11.3°C. On the other hand, the HFPCC also displays different radial temperature differences at different time points, measuring at 0.1°C, 4.3°C, 5.4°C, 5.7°C, 7.7°C, 10.5°C, 11.1°C, 10.6°C, and 9.7°C. Similar to BC, the maximum temperature difference occurs within a 24-hour period. Notably, the maximum temperature difference in the HFPCC is 35.84% lower compared to BC. This highlights the substantial capability of HFPCC in significantly mitigating the internal temperature difference within the concrete, consequently reducing the occurrence of temperature-induced cracks in the shaft wall structure.

## 3 Numerical simulation

To conduct numerical simulation, certain assumptions are considered, and the model of the frozen shaft wall is simplified into a two-dimensional plane problem.

1. It is presumed that the concrete is homogenous and exhibits isotropy.
2. The thermal conductivity and specific heat of the concrete are assumed to remain constant throughout the simulation.
3. The airflow temperature and the interfacial heat transfer coefficient are considered to be steady values.
4. It is assumed that the temperature field is uniformly distributed along the vertical direction.
5. It is presumed that the temperature of the freezing tube remains unchanged throughout the simulation.

### 3.1 Heat conduction differential equation of concrete

#### 3.1.1 Heat conduction equation of BC

The temperature field in the early hydration process of BC belongs to unsteady heat conduction with internal heat source.

The temperature at time *t* at a certain position is represented by *T*(*x*,*y*,*t*), and the corresponding two-dimensional heat conduction equation is shown in Eq (1).

$$\rho c\frac{\partial T}{\partial t}=\lambda (\frac{\partial^{2}T}{\partial x^{2}}+\frac{\partial^{2}T}{\partial y^{2}})+Q_{1}$$ (1)

Where *ρ* is the density of concrete, kg/m^3^, *c* represents the specific heat of concrete, J/(kg·°C), *λ* denotes the thermal conductivity, W/(m·°C), *t* represents the age, h, and *Q*_1_ is the internal heat source, W/m^3^。

The internal heat source term of the transient temperature field in the hydration heat release stage of concrete can be determined by measuring the adiabatic temperature increase, as illustrated in Eq (2) [34].


$$Q_{1}=c\rho \frac{\partial \theta (t)}{\partial t}=c\rho ab\theta_{0}t^{b-1}e^{-at^{b}}$$ (2)

The adiabatic temperature rise test results were fitted using Eq (2), yielding model parameters of a = 1.002×10^−5^ and b = 4.028.

#### 3.1.2 Heat conduction equation of HFPCC

During the initial stages of hydration in phase change concrete, the heat transfer mechanism enhances the phase change process. The corresponding heat transfer equation is shown in Eq (3).

$$\rho c\frac{\partial T}{\partial t}=\lambda (\frac{\partial^{2}T}{\partial x^{2}}+\frac{\partial^{2}T}{\partial y^{2}})+Q_{1}+Q_{2}$$ (3)

Where *Q*_2_ denotes the heat released per unit volume of phase change material per unit time per unit volume, W/m^3^.

Let the starting temperature of phase transition be *T*_*on*_, the ending temperature of phase transition be *T*_*end*_, and the latent heat of phase transition be Δ*H*, *Q*_2_ as shown in Eq (4).

$$Q_{2}=\rho \frac{\partial (\alpha \cdot \Delta H)}{\partial t}$$ (4)

Where *α* is liquid phase fraction, as shown in Eq (5).


$$\alpha =\{\begin{array}{ll} 0, & T<T_{on}\\ \frac{T-T_{on}}{T_{on}-T_{end}}, & T_{on}\leq T\leq T_{end}\\ 1, & T>T_{end} \end{array}$$ (5)

### 3.2 Modeling process

#### 3.2.1 Material parameter

The pouring temperature of the concrete is adjusted to 30°C, while the initial temperature of the frozen soil is -2°C. Additionally, the freezing pipe remains at a constant temperature of -2°C. Table 4 presents the physical characteristics of the material.

**Table 4:** Material parameters.

Material	Density (kg·m^-3^)	Thermal conductivity (W·m^-1^·°C^-1^)	Specific heat (J·kg^-1^°C^-1^)	Phase transition region (°C)	Latent heat (J·kg^-1^)
BC	2450	1.96	970	-	-
HFPCC	2352	2.10	1230	40~44	5000
Frozen soil	2028	2.10	1020	-1~0	7000
Thawed soil	2028	1.50	1520	-1~0	7000
Foam board	18	0.03	2000	-	-

#### 3.2.2 Establishment of geometric model

**Fig 10:**
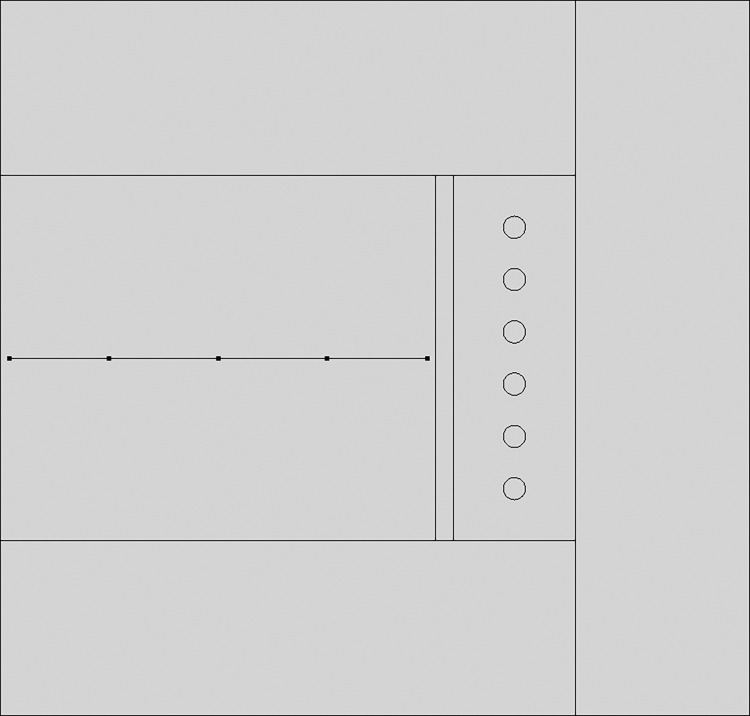
Geometric model diagram.

**Fig 11:**
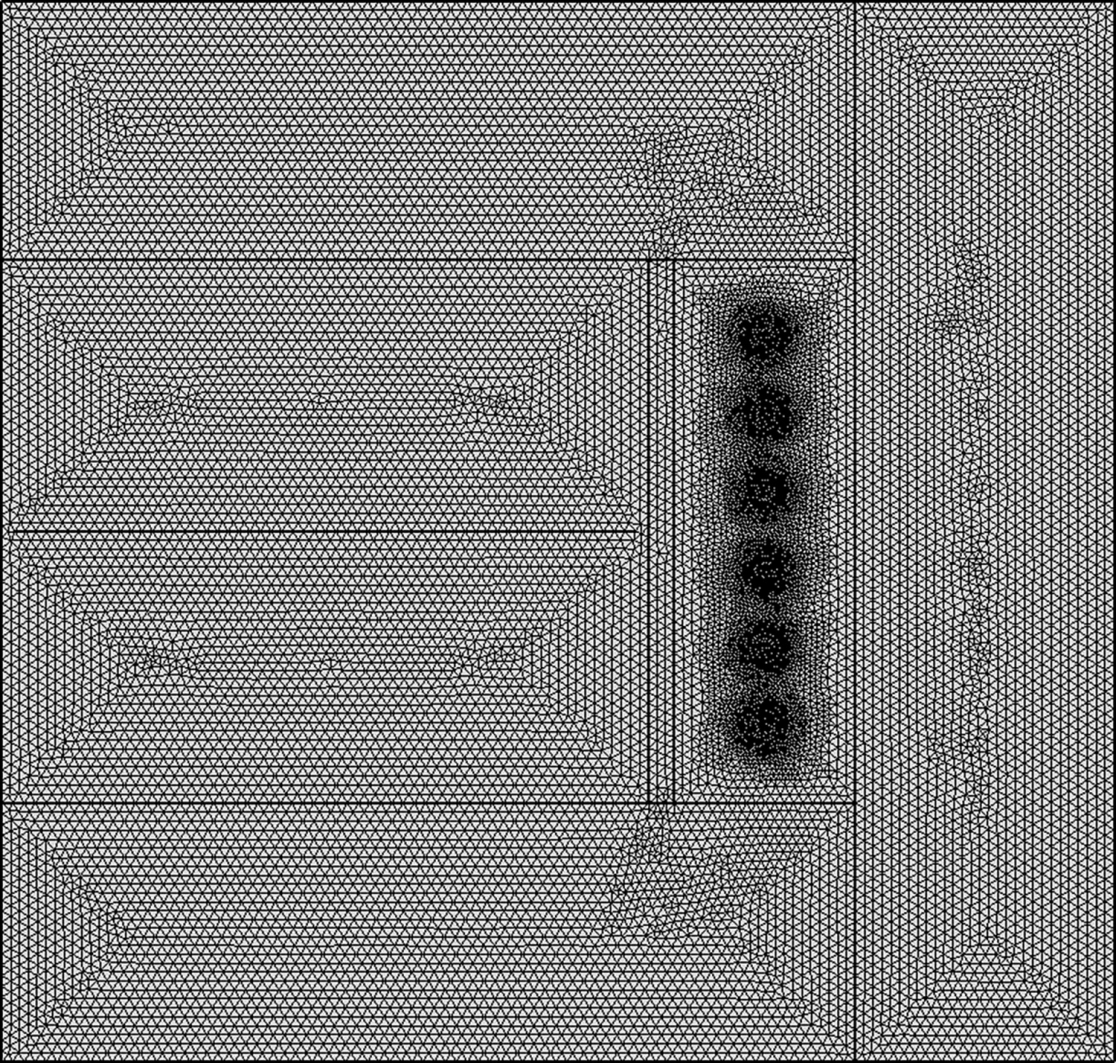
Model grid division.

Using COMSOL Multiphysics finite element software, numerical simulations were conducted to model the experimental setup described in Section 2, creating an accurate geometric representation of the frozen wellbore walls reinforced with hybrid fibers. This geometric model is further simplified into a two-dimensional form, as demonstrated in Fig 10. In order to achieve higher accuracy, the model was divided into a very fine mesh, with the complete mesh comprising 32,409 domain elements and 1,002 boundary elements, as shown in Fig 11.

#### 3.2.3 Boundary condition

The frozen shaft wall model test is positioned in direct contact with the surrounding air, and the third kind boundary condition is applied. The specific equations utilized are shown in Eq (6)–(7):

Left and right surface:

$$\pm \lambda \frac{\partial T}{\partial y}=h(T_{\mathrm{air}}-T_{\mathrm{side}})$$ (6)

Front and back surface:

$$\pm \lambda \frac{\partial T}{\partial x}=h(T_{\mathrm{air}}-T_{\mathrm{side}})$$ (7)

Where *T*_air_ is ambient temperature, *h* is coefficient of convective heat transfer. The ambient temperature is kept constant at 30°C, and its value is related to the wind velocity (*v*). The expression is shown in Eqs (8)–(9):

Foam board [35]:

$$h_{f}=11.63+7\sqrt{v}$$ (8)

Concrete [36]:

$$h_{c}=4.11+3.06v$$ (9)

### 3.3 Numerical simulation results analysis

#### 3.3.1 Validation of numerical simulation results

**Fig 12:**
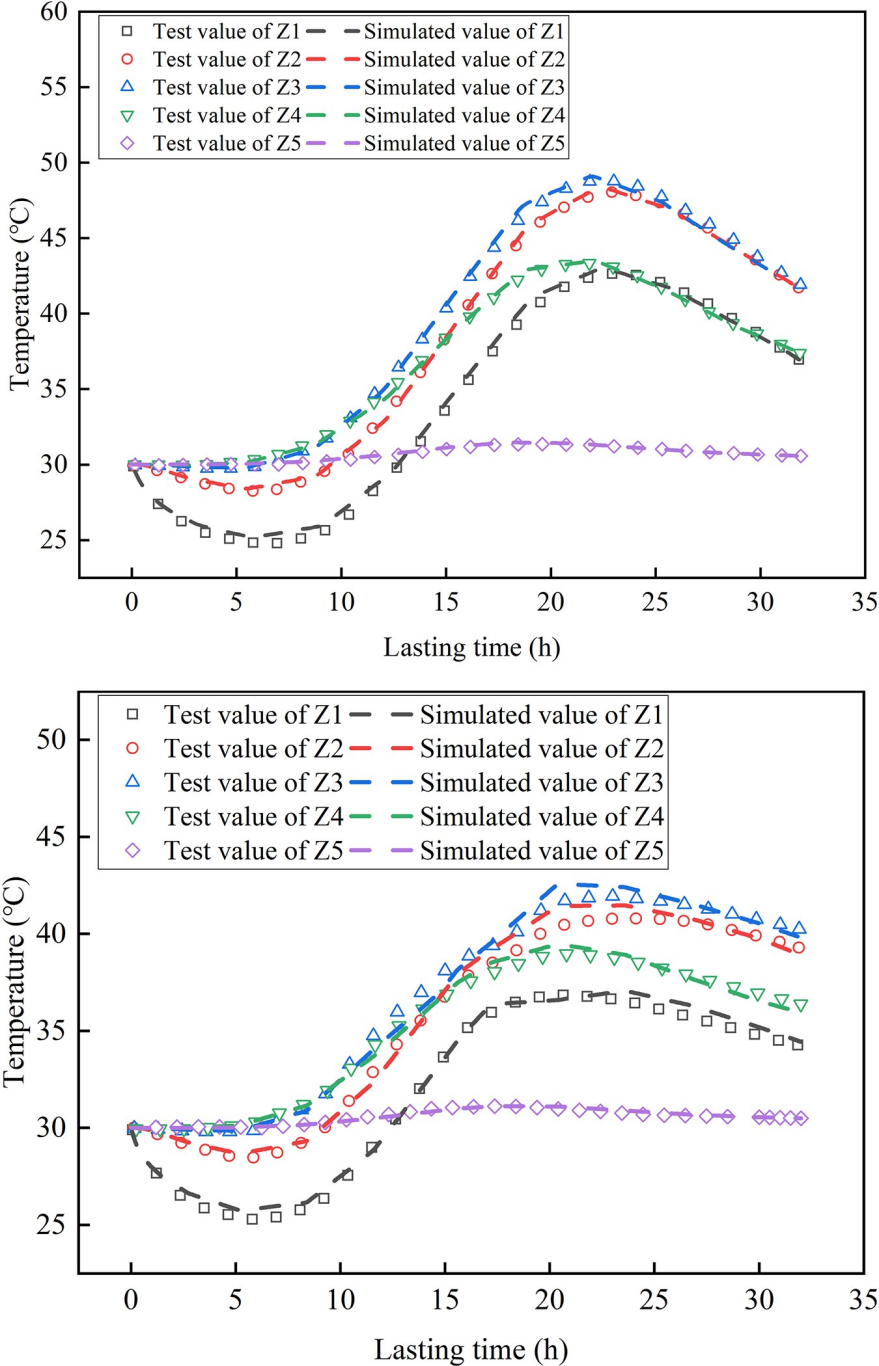
Comparison of test results and simulation results.

Fig 12 shows the test results and simulation results of each measuring point. After a span of 10 hours from the commencement of concrete pouring, the simulation outcomes typically exhibit higher readings compared to the test results, particularly at the measuring locations adjacent to the shaft wall. The reason behind this disparity is attributed to the decrease in temperature of the foam plate, which is influenced by the frozen wall. However, it is worth noting that the initial conditions of the model were not taken into account, contributing to the observed differences. Following this period, the simulation outcomes once again exhibit a remarkable concurrence with the experimental findings. The simulated maximum temperatures for Z1 to Z5 are 43.2°C, 48.3°C, 49.1°C, 43.6°C, and 31.5°C, respectively. The collective average discrepancy amounts to 3.51%.

During the initial stage of pouring concrete, it exhibits a comparable behavior to the BC. However, once it reaches the phase change temperature, a minor discrepancy arises between the simulation outcomes and the experimental findings. This divergence could potentially be attributed to the uneven dispersion of phase change aggregate. The collective average discrepancy amounts to 7.92%. In summary, the numerical model established in this paper can reliably simulate the heat transfer process of concrete freezing shaft wall.

#### 3.3.2 Distribution characteristics of wellbore temperature field

**Fig 13:**
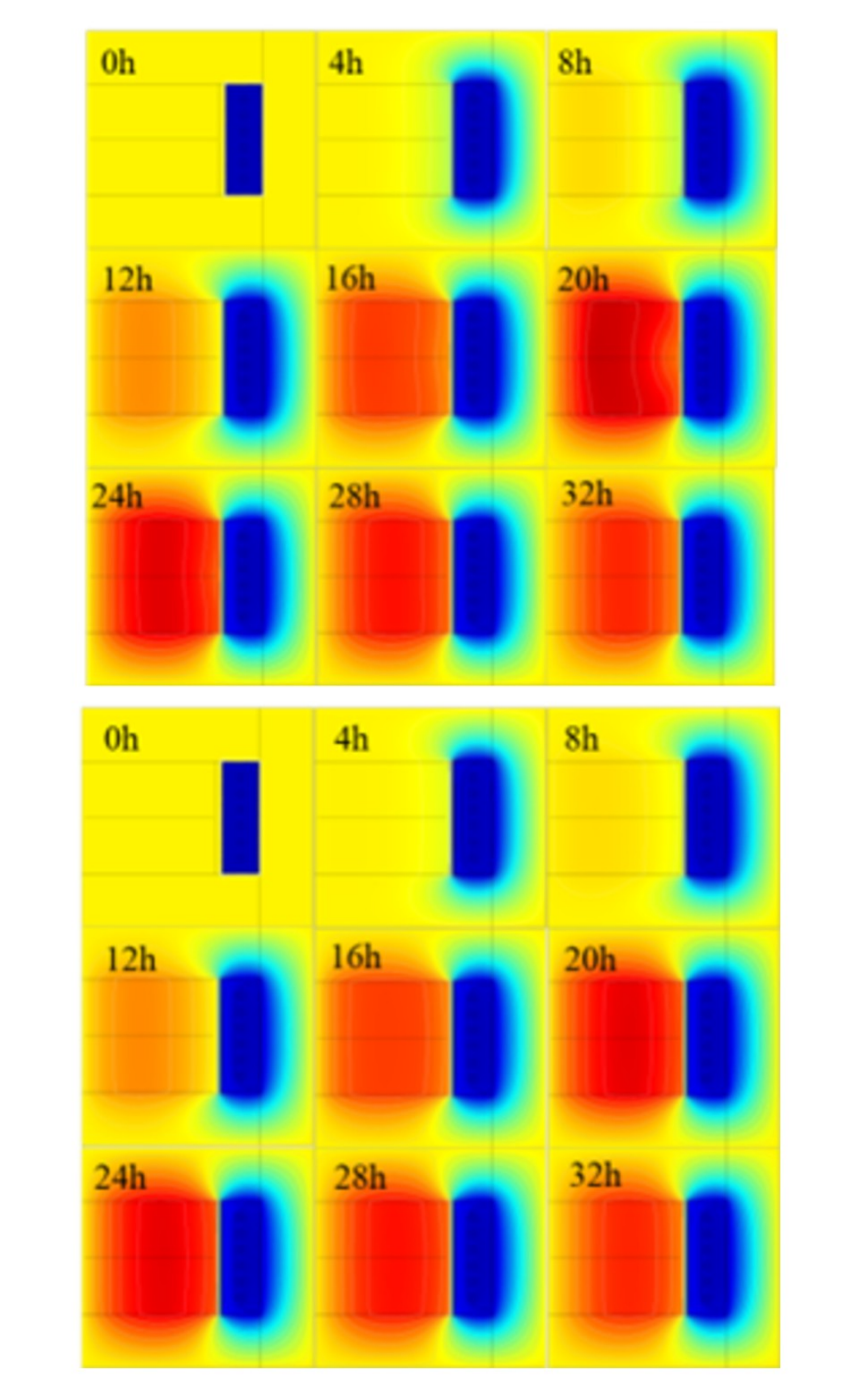
Temperature distribution of ordinary concrete at different times.

Fig 13 shows the temperature field distribution cloud diagram of shaft wall at different time. In the initial stage, the temperature shows a decreasing trend along the radial direction. Over time, the temperature assumes a "parabolic" form along the radial axis. Additionally, the highest temperature steadily shifts towards the frozen wall and eventually reaches a state of stability after 20 hours. The isotherm density in the wellbore increases first and then decreases with time, and reaches its peak density between 20 to 24 hours, signifying the occurrence of the highest radial temperature difference within the same time frame. The temperature distribution in HFPCC exhibits a comparable pattern to that of regular concrete within the initial 12 hours. However, beyond this time frame, the maximum temperature attained by HFPCC is notably lower than that of BC. Additionally, the rate at which isotherm density changes with time is slower in HFPCC compared to BC. The results indicate that phase change concrete experiences only a minor increase in temperature, with a slow temperature rise rate and smaller radial temperature difference. This further validates the practicality of utilizing hybrid fiber phase change concrete for frozen shaft walls.

## 4 The effect of parameters on the temperature field of borehole wall

### 4.1 The effect of phase transition temperature

**Fig 14:**
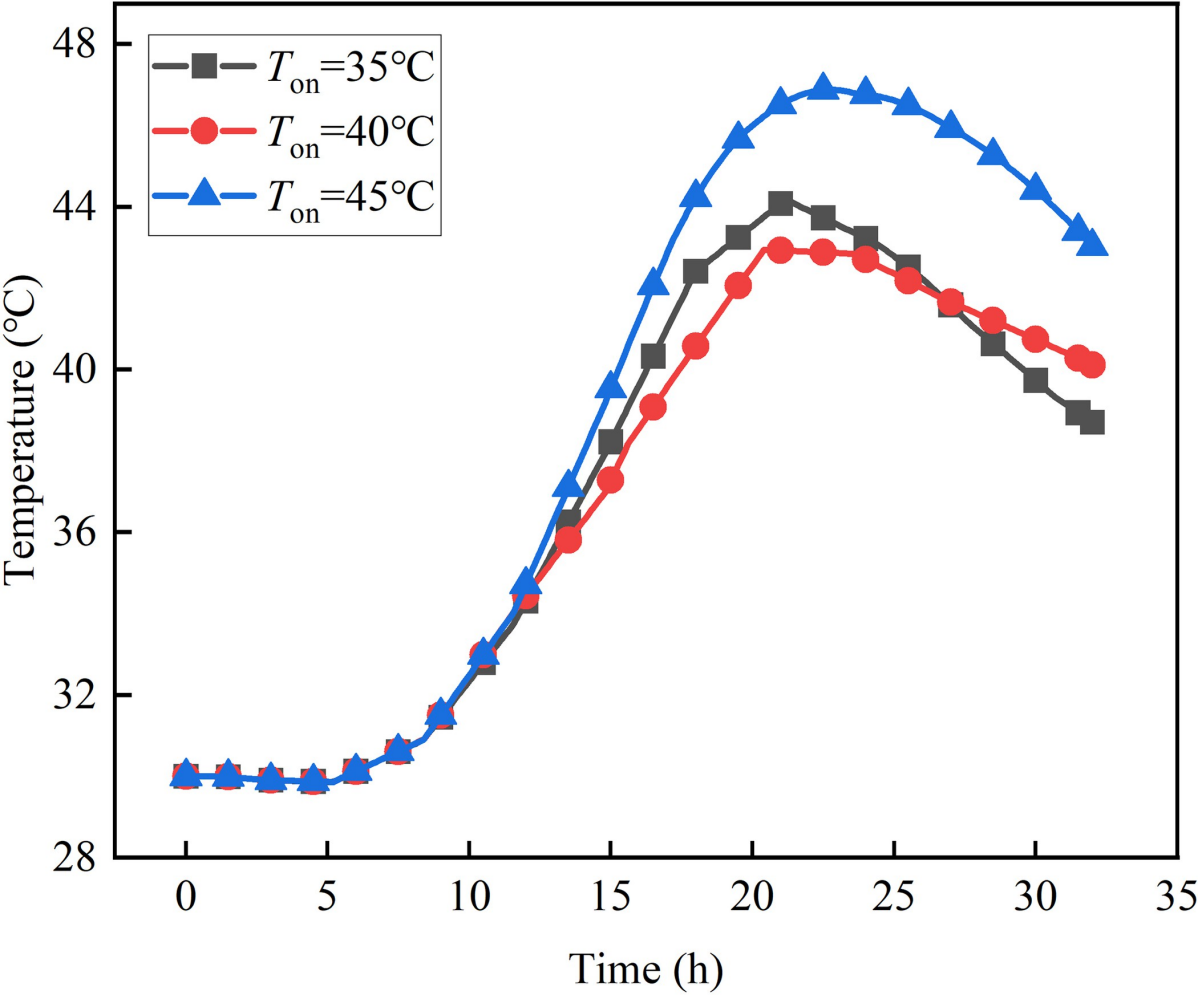
The law of temperature over time of measurement point Z3.

Fig 14 shows the variation of phase transition temperature Z3 with time. When *T*_*on*_ is 40°C, the Z3 exhibits the lowest peak temperature. However, when the *T*_*on*_ is either 35°C or 45°C, the peak temperature increases by 3.03% and 9.32% respectively compared to the *T*_*on*_ = 40°C. The heat absorbed during phase change inadvertently absorbs the ambient heat when the temperature at which phase change takes place is in proximity to the surrounding temperature, leading to an unnecessary loss of latent heat. Similarly, when the phase change temperature aligns with the peak temperature, the absorption of heat during phase change remains incomplete. Consequently, certain phase change materials fail to undergo the necessary change in state, resulting in a squandered amount of latent heat.

**Fig 15:**
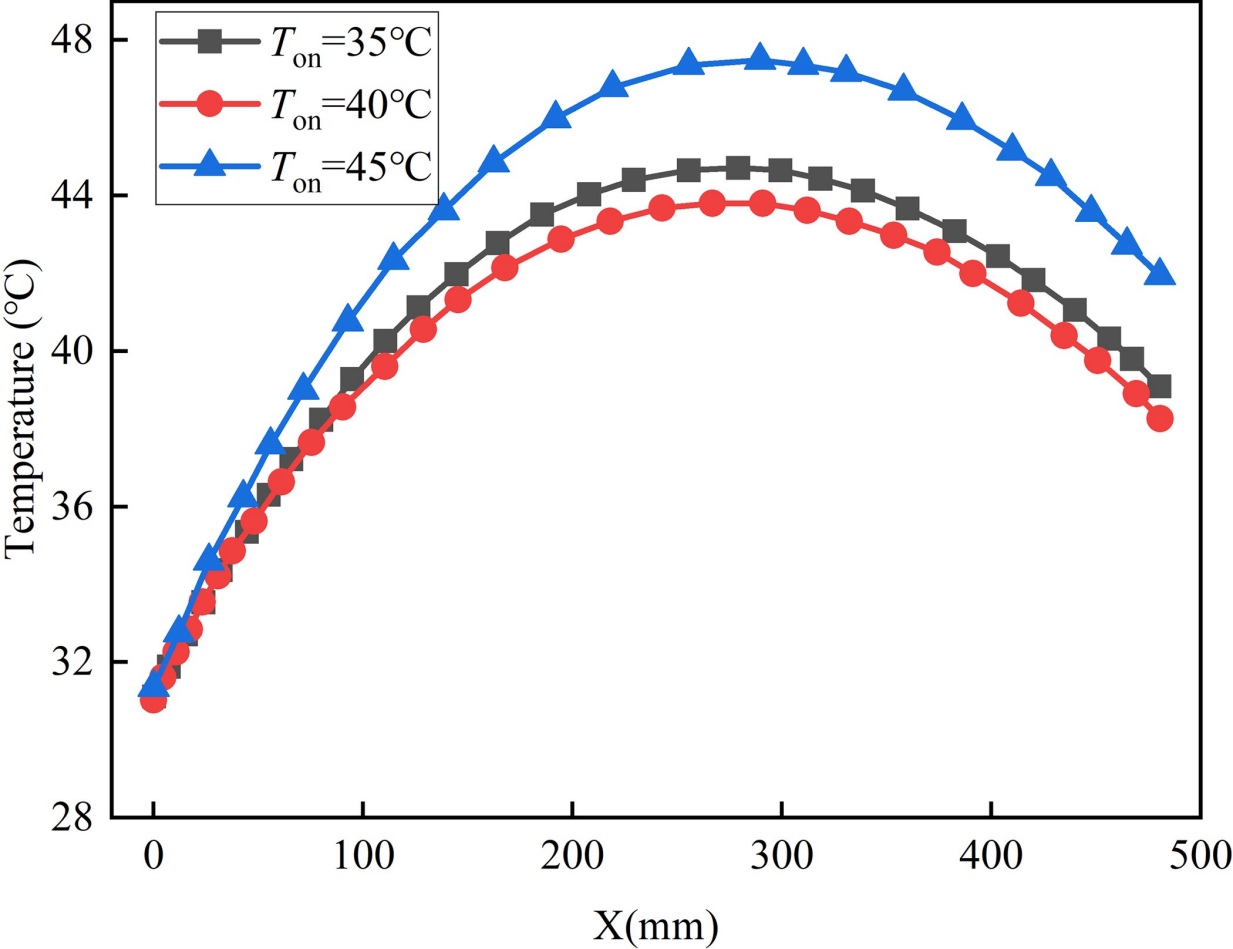
The temperature varies with X.

Fig 15 shows the curve of temperature changing with x when Z3 reaches the peak temperature. It is observed that the maximum temperature difference is the least when *T*_*on*_ = 40°C, amounting to 12.7°C. However, if *T*_*on*_ is set at 35°C or 45°C, the maximum temperature difference increases by 8.13% and 30.89% respectively, in comparison to *T*_*on*_ = 40°C. In summary, it can be seen that the phase change temperature has a great influence on the temperature field of the concrete shaft wall. The appropriate phase change temperature should be selected in practical engineering applications. In this paper, 40°C is more reasonable.

### 4.2 The effect of phase change latent heat

**Fig 16:**
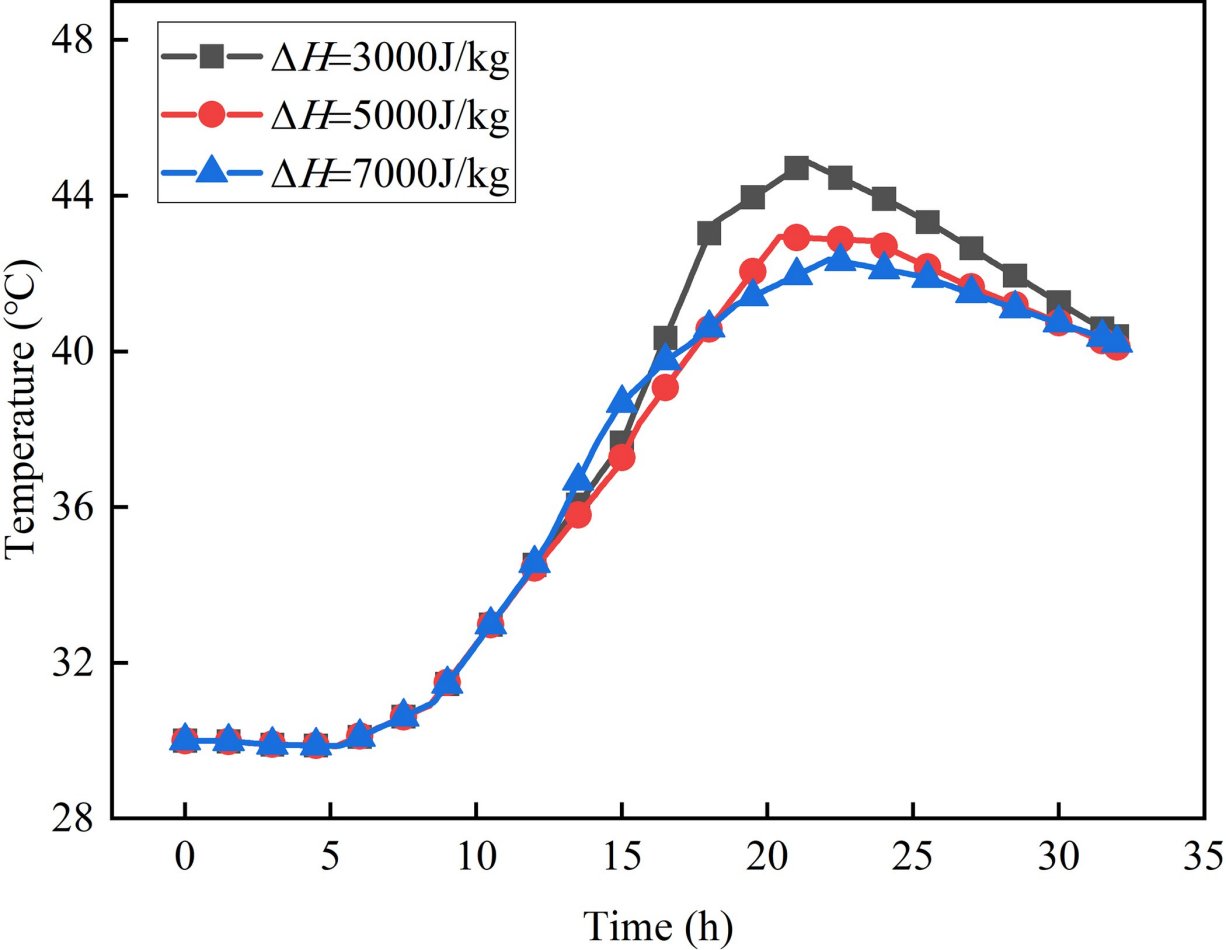
The law of temperature over time of measurement point Z3.

Fig 16 shows the variation of the temperature of different phase change latent heat Z3 with time. As the concrete attains the temperature for phase transition, it experiences a reduction in its peak temperature with the increase of latent heat of phase change. Additionally, the rate at which the concrete’s temperature alters starts to gradually diminish. This phenomenon can be attributed to the fact that a higher phase change latent heat implies a greater amount of heat absorbed during the phase change process, thereby mitigating the temperature rise during concrete hydration.

**Fig 17:**
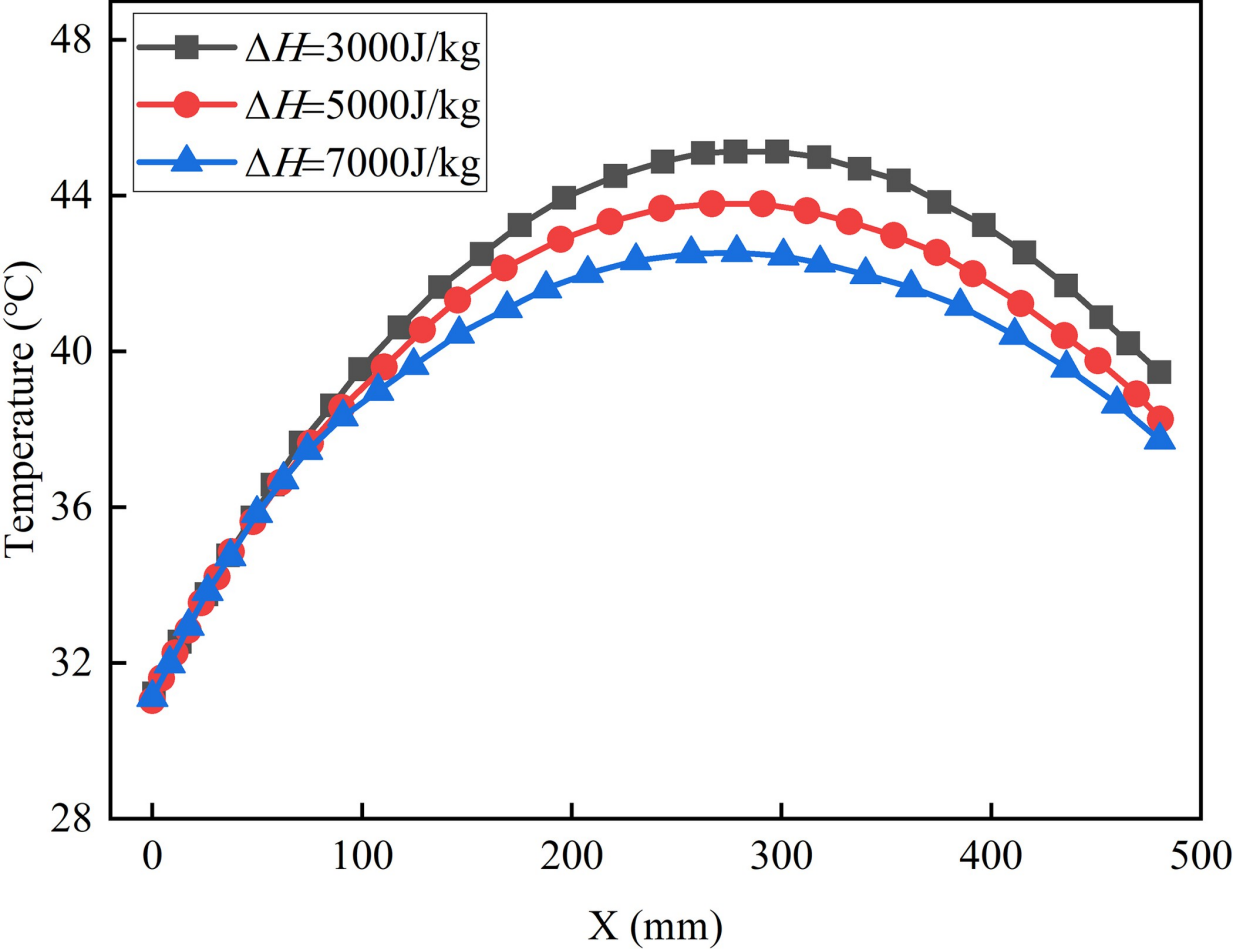
The temperature varies with X.

Fig 17 shows the curve of temperature changing with x. When Δ*H* is equal to 3000J/kg, the largest radial temperature variation reaches 14.0°C. However, when Δ*H* is set to 5000J/kg and 7000J/kg, the maximum temperature difference is 9.29% and 18.57% respectively lower compared to the scenario where Δ*H* equals 3000J/kg. To summarize, it is evident that the latent heat significantly impacts the temperature distribution within a concrete shaft wall. In engineering scenarios, it is advisable to prefer materials with a high latent heat of phase change whenever feasible. Additionally, it is crucial to enhance the energy storage and heat release capabilities of phase change materials by optimizing their preparation methods.

## 5 Conclusion

In this study, the evolution of the temperature field in shaft walls was investigated through numerical simulations and model experiments. The thermal control effects of hybrid fiber phase change concrete (HFPCC) applied to frozen shaft walls were also explored. Furthermore, it analyzes the influence of different phase change temperatures and latent heat on the distribution of the temperature field in the frozen shaft wall. The following conclusions were reached:

1. Following the pouring of the concrete shaft wall model, the temperature demonstrates a gradual decrease over time during the 0h to 6.9h period. Subsequently, within the 6.9h to 22.3h duration, the temperature experiences a rapid ascent, displaying the highest rate of increase. Beyond the 22.3h mark, the temperature undergoes a sharp decline. The peak temperature of HFPCC is generally lower than that of BC, with a noticeably shorter duration of high temperature rise. Following the peak temperature, the descent curve of HFPCC is more gradual.
2. The temperature distribution inside the shaft wall model forms a "parabolic" shape along the radial direction, with higher temperatures at the center and lower temperatures on both sides. As time progresses, the highest temperature continuously shifts towards the side of the frozen wall. HFPCC significantly reduces the temperature difference within the concrete, with a reduction of 35.84% in the maximum temperature difference along the radial direction compared to BC.
3. The phase transition temperature and latent heat of phase transition have a significant impact on the hydration temperature rise of concrete. In practical engineering, it is advisable to choose a moderate phase transition temperature. The peak temperature and temperature difference are directly proportional to the latent heat of phase transition. By increasing the latent heat of phase transition of the phase change material, the temperature control performance of phase change concrete can be further improved.

## Supporting information

S1 Table Measured data. (XLSX)
